# Glycerol monolaurate inhibition of human B cell activation

**DOI:** 10.1038/s41598-022-17432-4

**Published:** 2022-08-05

**Authors:** Micaela G. Fosdick, Shannon Loftus, Isabella Phillips, Zeb R. Zacharias, Jon C. D. Houtman

**Affiliations:** 1 Biomedical Sciences Graduate Program, Subprogram in Molecular Medicine, Carver College of Medicine, University of Iowa, 2110 MERF, Iowa City, IA 52242 USA; 2 Department of Microbiology and Immunology, Carver College of Medicine, University of Iowa, Iowa City, USA; 3 Human Immunology Core, Holden Comprehensive Cancer Center, Carver College of Medicine, University of Iowa, Iowa City, USA

**Keywords:** Lymphocyte activation, B cells

## Abstract

Glycerol monolaurate (GML) is a naturally occurring antimicrobial agent used commercially in numerous products and food items. GML is also used as a homeopathic agent and is being clinically tested to treat several human diseases. In addition to its anti-microbial function, GML suppresses immune cell proliferation and inhibits primary human T cell activation. GML suppresses T cell activation by altering membrane dynamics and disrupting the formation of protein clusters necessary for intracellular signaling. The ability of GML to disrupt cellular membranes suggests it may alter other cell types. To explore this possibility, we tested how GML affects human B cells. We found that GML inhibits BCR-induced cytokine production, phosphorylation of signaling proteins, and protein clustering, while also changing cellular membrane dynamics and dysregulating cytoskeleton rearrangement. Although similar, there are also differences between how B cells and T cells respond to GML. These differences suggest that unique intrinsic features of a cell may result in differential responses to GML treatment. Overall, this study expands our understanding of how GML impacts the adaptive immune response and contributes to a broader knowledge of immune modulating monoglycerides.

## Introduction

Glycerol monolaurate (GML) is a broad-spectrum antimicrobial agent, effective against fungi, enveloped viruses, gram positive bacteria, and select gram negative bacteria^1–6^. GML is a naturally occurring monoglyceride that is produced from triglycerides found in high concentrations in breastmilk and palm tree-oils such as coconut oil^7,8^. GML is used commercially in personal hygiene products and food items as a preservative. GML is also used as a homeopathic agent and is being tested for clinical applications, such as preventing HIV transmission, treating bacterial vaginosis, chronic sinusitis, acne, and preventing Toxic Shock Syndrome^4,9,10^ .

In addition to GML’s impact on microbes, recent studies have shown GML suppresses PBMC proliferation and inhibits T cell receptor (TCR) dependent T cell activation^3,11,12^. These studies have extensively analyzed how GML impacts several key signaling events in T cell activation. GML significantly alters T cell membrane dynamics by increasing of the amount of ordered and disordered lipid domains^12^. Disrupting the dynamics of these regions alters the cell’s ability to stabilize Linker for Activation of T cells (LAT) and PLCγ clustering, and decreases AKT and PI3K signaling^12^. Inhibition of protein clustering upon stimulation suppresses TCR dependent T cell activation and inhibits cytokine production^12–14^. Furthermore, GML alters cytoskeleton arrangement in T cells via disruption of the Arp2/3 complex that reduces cell adhesion and causes dysregulated filopodia formation^13^.

B cells also play a significant role in the adaptive immune system by producing antibodies needed for the prevention and elimination of infections. The primary activating receptor for B cells is the B cell antigen receptor (BCR) that binds directly to its antigen. Upon antigen binding, the BCR subunits are phosphorylated by Src-family protein kinases. This leads to the phosphorylation of signaling molecules such as Bruton’s Tyrosine Kinase (BTK), phospholipase Cγ2 (PLCγ2), and B cell linker protein (BLNK), which form the BCR signalosome. Src kinases also phosphorylate the costimulatory transmembrane protein CD19, which then activates the Phosphoinositide 3-kinase (PI3K)-protein kinase B (AKT) signaling pathway^15^. Initial activation is also impacted by the signaling of other co-stimulatory molecules, such as CD40 and CD86, that allow B cells to engage with T cells and will contribute to proliferation and differentiation of naïve B cells into functional antibody producing cells and memory cells^16–20^. Transmembrane protein CD40 is an early co-stimulatory molecule that is present before BCR activation and interacts with CD40 ligand (CD154) on T cells that contributes to increased BCR activation signaling. Conversely, CD86 is a later activation marker that is the ligand for CD28 on T cells that contributes to survival signaling and differentiation of B cells^18,20–22^. CD69 is expressed in both B and T cells, is one of the earliest cell surface markers expressed after antigen receptor activation, is primarily associated with lymph node retention, and increases the likelihood of B cell and T cell interaction^23–25^. Actin rearrangement and membrane dynamics also play a key role in effective B cell activation. Upon BCR interaction with antigen, altered actin regulation pathways drive clustering of BCR signaling proteins^26,27^; such as PLCy2 that regulate actin association with the plasma membrane, calcineurin that affects F-actin severing, and CD19-BTK that is associated with F-actin polymerization regulated by ARP 2/3 complex^27^. Actin rearrangement driven by the Arp 2/3 complex is utilized in B cells to stabilize immune synapse^26,28^. Ordered-phase like domains stabilize BCR micro-clusters aid in and stabilize protein clustering during activation^19,29–31^.

There are many similarities in the signaling pathways that are activated in both T and B cells. Upon TCR or BCR stimulation, protein clustering is induced to amplify intracellular signaling, including enhanced PI3K-AKT pathway, increased calcium signaling, and altered cytoskeleton rearrangement. However, B cells differ from T cells in key signaling proteins. LAT and SLP76 are essential in early T cell signaling; the B cell corollary to these signaling proteins is BLNK, which facilitates the formation of protein clusters^32^. Furthermore, B cells’ transmembrane protein CD19 serves a similar function as transmembrane CD28 in T cells, essential in the activation of the PI3K-AKT pathway^15,33,34^. While both lymphocytes undergo calcium signaling, T cells use PLCγ1 while B cells use PLCγ2 to induce calcium influx. These differences contribute to overlapping and unique activation events in B cells compared to T cells.

While GML’s impact has been described in T cells, no studies have examined how other immune cell types respond to GML treatment. To better understand how GML impacts other cells central to the adaptive immune response, we investigated how GML affects B cell activation. We explored several critical steps in B cell activation, including signaling protein phosphorylation, protein clustering and signalosome formation, cytokine production, and expression of cell surface markers. GML inhibits B cell cytokine production and early signaling events. In addition, GML induces dysregulated filopodia formation and changes in membrane dynamics in human B cells. Thus, we observed that GML inhibits B cell activation with a similar yet distinct mechanism as described previously in T cells. These data show that GML can inhibit activation of both classes of lymphocytes and may have a broader impact on the immune response.

## Results

### Glycerol monolaurate suppresses cytokine production and cell surface activation markers

**Figure 1:**
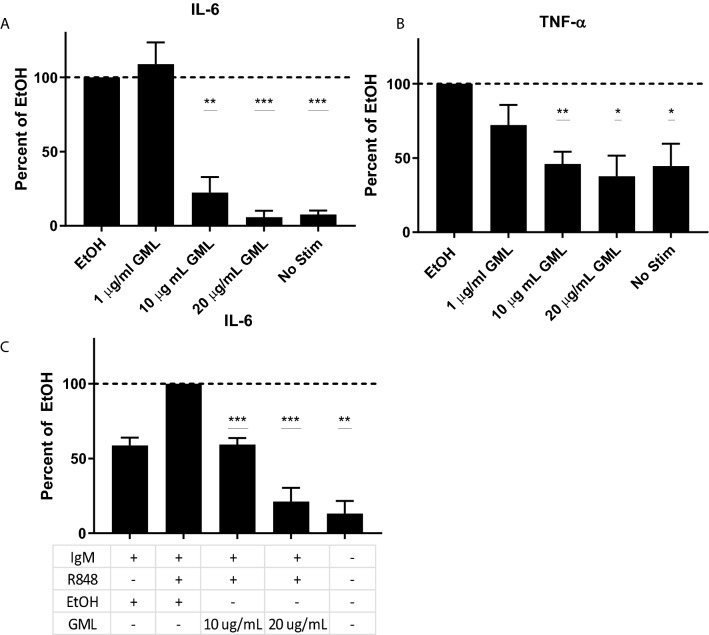
Glycerol Monolaurate inhibits BCR dependent and independent induced cytokine production. Primary human B cells were treated with 1, 10, or 20 ug/mL GML, or EtOH. Cells were stimulated overnight with 5 ug anti-IgM antibody and 20 ng soluble IL-4. Supernatants collected were analyzed via ELISA to determine levels (**A**) TNF-α and (**B**) IL-6. (**C**) Independent BCR induced IL-6 production was tested with the addition of a TLR7 agonist, R848. Data presented is normalized to the positive EtOH control, represented by the dotted line, and collected from 5 distinct donors. One-Sample T-test with theoretical value 1 was used to determine statistical significance. *p < 0.05, **p < 0.01.

Previous studies have shown that GML significantly inhibits cytokine production in TCR-stimulated T cells^12^. To get a broader understanding of how GML impacts other immune populations, the effect of GML on B cell function was evaluated via BCR-induced cytokine production. We treated primary B cells isolated from PBMCs with varying dosages of GML. These cells were then stimulated overnight with plate bound anti-IgM antibody and soluble Interleukin-4 (IL-4). GML treatment had no impact on cell viability (Supplemental Fig. 1). Supernatants were tested for Interleukin-6 (IL-6) and Tumor Necrosis Factor-alpha (TNF-α) by ELISA. Both the 10 ug/mL and 20 ug/mL treatment doses of GML significantly reduced BCR-induced IL-6 and TNF-α cytokine production (Fig. 1A,B). It is important to note that while TNF-α levels are higher than expected at baseline, GML treatment reduced TNF-α cytokine levels to baseline levels produced in non-stimulated B cells. We further investigated how GML would affect B cell activation with the addition of BCR independent stimulation with the Toll like receptor-7 (TLR7) agonist R848 (Fig. 1C). Interestingly, treatment with GML significantly inhibited IL-6 cytokine production in R848- co-stimulated B cells compared to vehicle controls.

**Figure 2:**
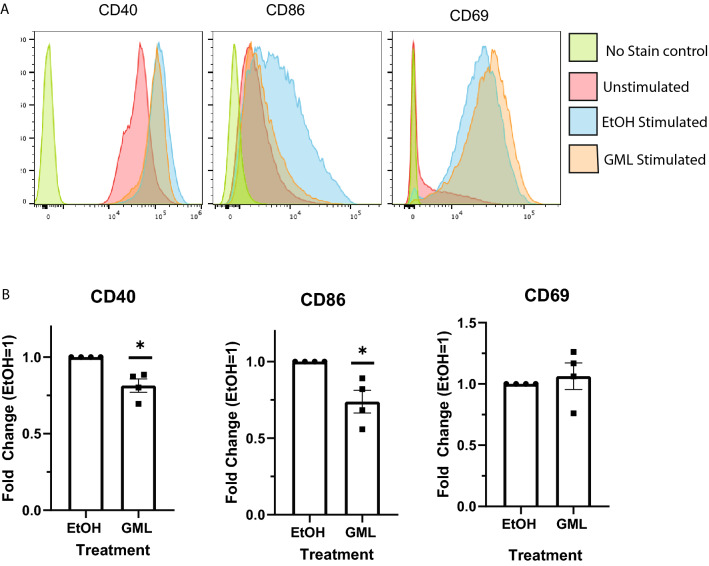
GML suppresses cell surface expression of survival costimulatory molecules. Primary human B cells were treated with 10 ug/mL GML or EtOH. Cells were stimulated for 24 h with 5 ug anti-IgM antibody and 20 ng soluble IL-4. After activation, cells were stained and analyzed on a Cytek Aurora. (**A**) Representative image of a single sample of data analyzed using FlowJo™ Software for Mac Version 10.8.1. Ashland, OR: Becton, Dickinson and Company; 2022 and Spectroflo Software for PC Version 3.0.3. Fremont, CA: Cytek Biosciences; 2022. (**B**) Geometric Mean Fluorescence Intensity of samples were normalized to EtOH control group. Graphs represent compiled date from 5 experimental replicates. The statistical significance was determined using a one-sample T-test with a theoretical value 1. *p < 0.05.

In addition to cytokine production, the effect of GML on cell surface markers was also tested. B cells were treated with GML or EtOH control and activated using anti-IgM antibody and soluble IL-4 for 24 h. Cells were then stained for CD86, CD40, and CD69 and analyzed using flow cytometry (Fig. 2A,B). Interestingly, these cell surface markers of activation were not impacted to the same degree as cytokine production. Cells treated with GML had significantly reduced CD86 and CD40 surface expression compared to cells treated with vehicle control (Fig. 2B). However, GML treatment had no impact on CD69 cell surface abundance (Fig. 2B). These data suggest while GML suppresses cytokine production and reduces expression of some cell surface markers, GML may have varying effects along the activation pathway.

### Glycerol monolaurate alters B cell membrane dynamics

**Figure 3:**
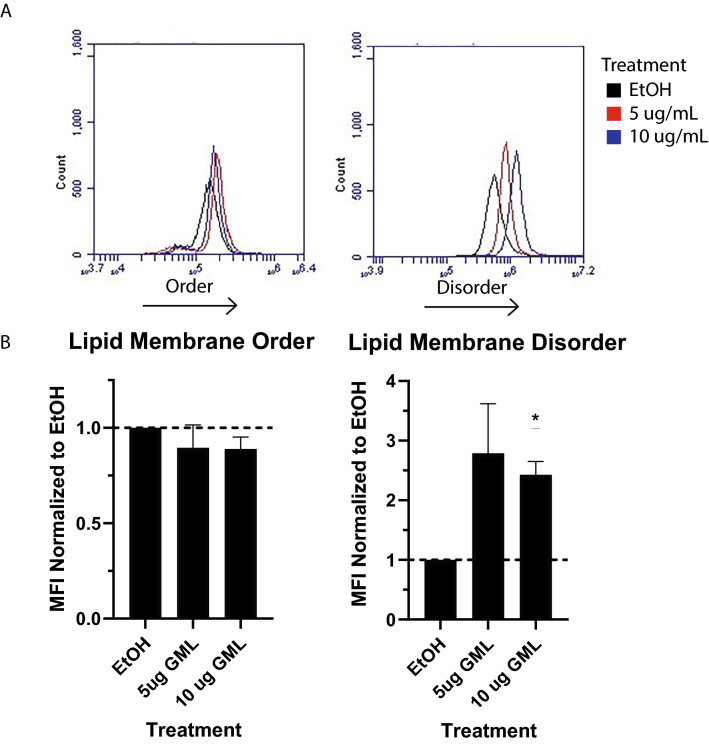
GML increases disorder in B cell membrane dynamics. B cells were pre-treated with various concentrations of Glycerol Monolaurate for 10 min. Cells were then stained using Di-4-ANEPPDHQ and analyzed by flow cytometry. Membrane order was measured using FL1 channel and Disordered membrane domains were measured using the FL3 channel. (**A**) Representative flow plot for each channel measured. (**B**) Mean fluorescent intensities averaged from 4 experiments with distinct donors. One Sample T-test with theoretical value 1 was used to determine statistical significance. *p < 0.05.

GML is thought to increase the levels of both ordered and disordered regions of the membrane via incorporating into the membrane and preventing free diffusion of lipids into and out of ordered regions^12^. Importantly, lipid membrane dynamics and lipid microdomains play a distinct role in B cell activation^19,29,35,36^. To examine the impact of GML on lipid dynamics, B cells were stained using the order/disorder sensing membrane dye Di-4-ANEPPDHQ (Fig. 3A). After treating primary B cells for 10 min with varying concentrations of GML, cells were stained with Di-4-ANEPPDHQ and analyzed using flow cytometry. Order and disorder were normalized to untreated cells. B cells treated with GML had a significant dose dependent increase in membrane disorder compared to vehicle treated controls (Fig. 3B). However, there were no significant changes detected in ordered membrane regions when compared to vehicle control (Fig. 3B). These data suggest that GML may disrupt B cell signaling through changes lipid membrane dynamics, although it appears to act with a different mechanism than its effects on membrane domains in T cells.

### Glycerol monolaurate inhibits BCR-induced phosphorylation

**Figure 4:**
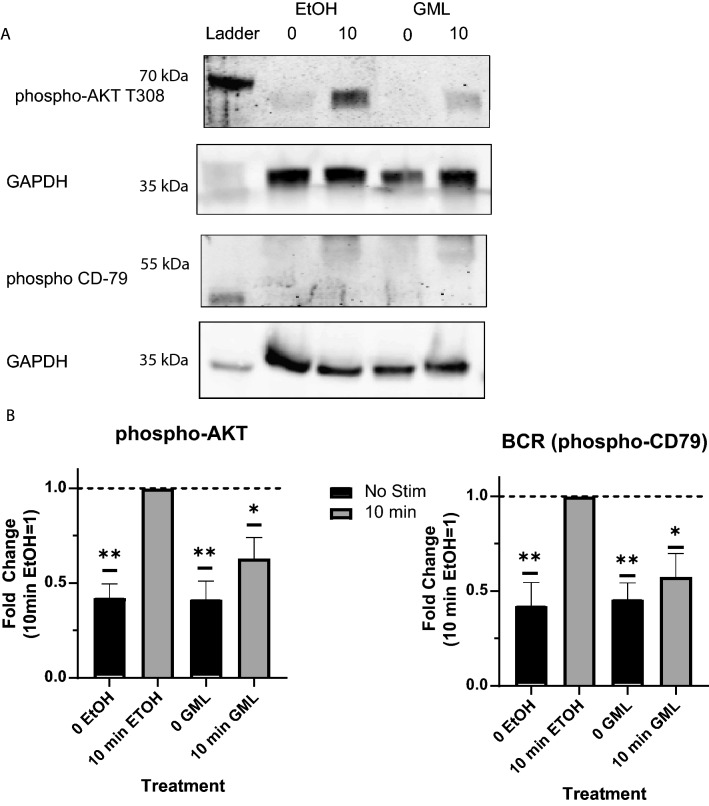
GML suppression of BCR induced phosphorylation of key signaling proteins. Cells were treated with EtOH or 10 ug/mL GML, stimulated with 5 ug/mL soluble anti-IgM antibody for 10 min. Cell lysates were tested using Western Blot analysis to determine phosphorylation of CD79 sub-unit of the BCR and T308 AKT. Blots were imaged and quantified using a Licor Odyssey imaging system. Samples were normalized to GAPDH and analyzed as a fold change with stimulated EtOH control set to 1. Data presented is 5 distinct donors compiled and analyzed using One-Sample T-test with theoretical value 1. *p < 0.05.

BCR dependent activation leads to phosphorylation of key signaling proteins, such as CD79 and AKT, which are needed to transmit membrane activation events across the cytoplasm to the nucleus. To determine how GML affected B cell signaling during activation, primary human B cells were pre-treated with GML or EtOH control and activated for 10 min using anti-IgM. Cell lysates were analyzed via quantitative immunoblotting. Phosphorylated CD79, a subunit of the BCR, was used as a measure of early signaling, while phosphorylated AKT was used as a surrogate for downstream signaling. Vehicle control treated cells responded to stimulation with unstimulated vehicle control treated cells exhibiting significantly less CD79 and AKT phosphorylation than stimulated vehicle control treated cells (Fig. 4A,B). Cells treated with GML had reduced responses to BCR stimulation, with significantly less phosphorylation of both CD79 and AKT in GML treated cells compared to vehicle control treated cells (Fig. 4A,B). Inhibition of CD79 and AKT phosphorylation in GML-treated B cells demonstrates GML inhibits early BCR dependent signaling cascades.

### Glycerol monolaurate inhibits BCR induced protein signalosome formation

**Figure 5:**
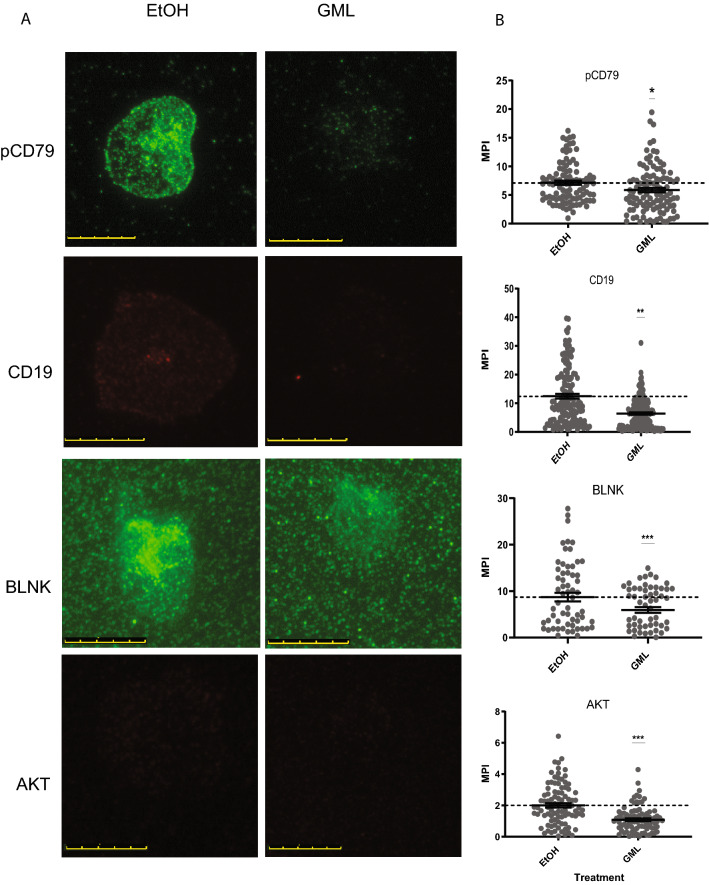
GML inhibits BCR induced signalosome formation. Cells were pretreated with 10ug/mL GML or EtOH control and stimulated for 10 min. on chamber glass slides coated with anti-IgM antibody. After stimulation, cells were fixed, permeabilized, and stained with anti-phosphorylated CD79, total anti-CD19, anti-phosphorylated BLNK, or total anti-AKT specific anti-bodies. Data presented was compiled from 3 experimental replicates. (**A**) Protein clusters were imaged using TIRF microscopy. Scale bar in each image represents 10 µM. (**B**) Images were analyzed measuring MPI in ImageJ. Statistical significance was calculated using a T-test. *p < 0.05, **p < 0.01, ***p < 0.001.

Changes in phosphorylation of CD79 and downstream AKT led to the question of how GML impacts earlier signaling events. B cell activation through the BCR induces protein clustering required to transmit signals to the cytoplasm^15,29,30^. To investigate how GML affects this phenomenon in B cells, TIRF microscopy was used to image protein clustering at or near the membrane. Cells were pre-treated with GML or EtOH as a vehicle control and stimulated with plate bound anti-IgM antibodies for 10 min. The cells were fixed and permeabilized and then stained for phosphorylated CD79, total CD19, phosphorylated BLNK, or total AKT using specific antibodies. Protein clusters were imaged using TIRF microscopy and analyzed by ImageJ. Compared to EtOH treated controls, GML treated cells had slight but significantly decreased pCD79 (BCR) and CD19 clustering, suggesting that GML impacts the clustering of the BCR after stimulation (Fig. 5A,B). BCR-mediated pBLNK and AKT clustering were also significantly decreased compared to EtOH controls (Fig. 5A,B). Interestingly, pBLNK and AKT clustering appeared to be more potently suppressed by GML treatment than BCR clustering with a larger difference in average MPI between GML treatment groups and vehicle control treatment groups observed for pBLNK and AKT compared to BCR components (Fig. 5A,B). Together, these data demonstrate GML alters early protein clustering downstream of BCR activation.

### Glycerol monolaurate alters B cell cytoskeleton rearrangement

**Figure 6:**
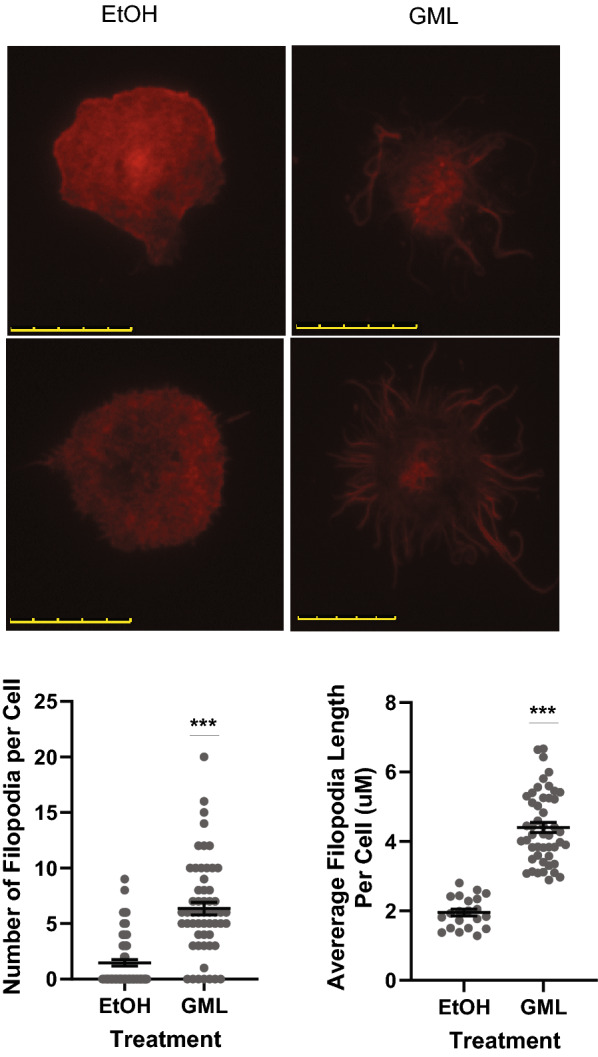
GML alters B Cell cytoskeleton arrangement. Cells pretreated with 10 ug/mL GML or EtOH control were stimulated for 10 min on chamber glass slides coated with anti-IgM antibody, fixed, and permeabilized. Actin was stained with Phalloidin and imaged using TIRF microscopy. Images were analyzed by counting number of filopodia per cell and measuring filopodia length in ImageJ. Data compiled from 2 distinct donors. T-test was used to calculate statistical significance. ***p < 0.001.

The activation of ARP2/3 complex is necessary for the controlled filopodia formation needed for the orientation and migration of B cells through tissues, in addition to enhancing immune synapse formation^26,27^. GML has been shown to disrupt TCR-induced regulation of ARP2/3 complex in human T cells and induce dysregulated filopodia formation^13^. To investigate how GML affected B cell cytoskeleton rearrangement, primary human B cells were pretreated with 10 ug/mL GML and plated on an anti-IgM antibody coated chamber cover slips. Filamentous actin was quantified by staining with FITC-labeled phalloidin followed by TIRF microscopy. The average number and length of filopodia per cell was measured using ImageJ. Cells treated with GML had significantly more filopodia per cell and a significant increase in filopodia length compared to vehicle control treated cells (Fig. 6), suggesting that GML treatment dysregulates cytoskeleton rearrangement in B cells.

## Discussion

GML is known to inhibit TCR dependent T cell activation^12,13^. However, to better understand potential clinical uses of GML, it is important to understand if GML affects other cell types. In this study we explored how GML more broadly impacts the adaptive immune response by testing how it affects primary human B cells. Treatment with 10 ug/mL or above of GML resulted in an increase in membrane disorder and inhibits BCR dependent phosphorylation signaling cascades. As a consequence of disrupting these signaling cascades, GML treatment results in a significant decrease in signalosome formation/protein clustering, suppression of select activation cell surface markers, and ultimately cytokine production. Furthermore, treating primary human B cells with GML results in filopodia protrusions not seen in vehicle treated cells. Ultimately, this study conclusively shows that GML inhibits human B cell activation. 

There is a clear similarity between the impact of GML on B cells and T cells, with the suppression of cytokine release, decrease in protein clustering, and changes in lipid membrane dynamics suggesting a similar mechanism of action^12–14^. Interestingly, there are also noticeable differences in how GML affects these lymphocytes. Broadly speaking, GML appears to have a more severe impact on B cell signaling pathways when compared to its effects on TCR signaling. For example, in T cells, GML does not inhibit phosphorylation of Linker for Activation of T cells (LAT) but does inhibit clustering of phosphorylated LAT^12^. In B cells, we not only see a significant decrease in phosphorylated BLNK clustering, but also a reduction of the phosphorylation of BLNK. One potential reason for this observation is the stimulation of IgM+ B cells, which are largely naïve B cells. In contrast, the T cells used in prior studies were previously activated before experimentation. While rested, this is considered an effector T cell, which are more sensitive to stimulation and can be activated using up to 10 times less antigen than what is necessary for naïve responses^37,38^. Sensitivity to stimulation may also explain the differential impact GML had on CD69 cell surface expression compared to CD86 and CD40. Interestingly, although CD40 and CD86 cell surface expression was suppressed by GML, the earliest cell surface marker for activation, CD69, was not affected by GML treatment. This may be a result of timing or a result of CD69 being more sensitive to lower doses of stimulation. However, while CD69 is the earliest marker of activation, CD86 and CD40 expression allow engagement with T cells and are required for the survival, proliferation, and activation of B cells^16–19^.

This increased impact of GML on signaling in B cells could also be a result of the second key difference we discovered, specifically changes in membrane dynamics. When we analyze changes in membrane dynamics in B cells, there is a significant increase in the amounts of disordered lipid domains while ordered lipid domains are not substantially altered. This change in lipid membrane dynamics differs from the pattern described previously for T cells, where GML induces an increase in both ordered and disordered lipid domains^12^. One potential explanation for this is the baseline of a B cell’s ordered regions. The association of the B cell receptor with lipid rafts varies depending on developmental and activation status of B cells^36,39,40^. It is possible that by using naïve B cells, we would not see as drastic changes to the ordered regions because they are smaller and more dispersed than in previously activated T cells. Interestingly, when testing naïve T cells, we saw a similar phenomenon with less of an increase in membrane ordered domains than seen previously in Th0 cells (data not shown). Several studies have shown lipid rafts and microdomains present in naïve cells increase in size and number after activation^19,29,36,41,42^. Alternatively, this could be evidence that B cells are more sensitive to membrane changes and the sharp increase in disordered lipid domains was enough to inhibit the early signaling phosphorylation. These differences in responses in B cells, suggest unique features of a cell and its activation status may result in differential responses to GML treatment in other cell types as well.

In conclusion, this study demonstrates that GML effectively inhibits BCR-dependent B cell activation. This expands our understanding of how GML impacts the immune response on a broader scale. These studies suggest clinical use of GML would inhibit adaptive immune response by suppressing both B cell and T cell responses. This may shift the clinical use of GML from anti-microbial to an immune-suppressive compound that can potentially be used to prevent adaptive immune responses at cutaneous or mucosal surfaces, or sensitization to other active ingredients in topical products. Furthermore, these results further confirm that the mechanism of action of GML is not T cell specific, suggesting that it may impact other immune cells beyond B cells and T cells. These studies lay the foundation for future studies examining the effects of GML on the immune response, an area of interest for future research. Differences seen in this study suggest immune cells that are more sensitive to membrane changes may be more severely affected by GML treatment. In addition, our work suggests that GML may impact the function of non-immune cell types involved in the immune response, such as epithelial cells, that are found at sites of potential GML treatment. Overall, this study expands our understanding of how GML impacts the adaptive immune response and contributes to a broader understanding of the application of immune modulating monoglycerides.

## Materials and methods

### Primary human B cell isolation

PBMC’s were isolated from leukocyte reduction cone obtained from Degowin Blood Center at the University of Iowa Hospital and Clinics. Donors are anonymous and have provided written informed consent to allow their cells that are normally discarded to be used in research studies. The recruitment protocol and written informed consent document were approved by the Institutional Review Board for the University of Iowa. All samples were provided to investigators de-identified. Therefore, further IRB approval for the use of the cells by the investigators was not needed based on Federal Regulation 46.101.B4. All experiments were performed in accordance with approved guidelines. PBMC’s were isolated via density gradient using Lymphoprep (StemCell Technologies 07801). B cells were isolated from PBMCs using a negative selection Human B Cell Isolation kit (StemCell Technologies 17954) following the provided protocol. B cells were rested at 37 °C in Complete RPMI 1640 media until used and used within 5 h of negative selection.

### enzyme-linked immuno assay detection of cytokines

B cells were treated with various concentrations of GML or 0.009% EtOH as a vehicle control treatment in serum free RPMI 1640 media. They were stimulated overnight with plate-bound 5 ug/mL IgM anti-body (Jackson ImmunoReserach 109-006-129) and 20 ng/mL soluble recombinant IL-4 (BioLegend 574004). In addition, B cell receptor independent co-stimulation was tested using the addition of 5ug/mL TLR7 agonist R848 (Enzo ALX-420-038). Concentration of secreted IL-6 in the supernatant was detected via standard TMB ELISA using purified anti-human IL-6 antibody (BioLegend 501102) to capture and Biotin IL-6 (Biolegend 501201) for detection. Data presented is normalized to the positive EtOH control and collected from 3 to 5 distinct donors. One-Sample T-test with theoretical value 1 was used to determine statistical significance.

### Cell surface marker expression

B cells were treated with 10 ug/mL GML or 0.009% EtOH as vehicle control treatment in serum free RPMI 1640 media. They were stimulated for 24 h with 5 ug/mL anti-IgM antibody (Jackson ImmunoReserach 109-006-129) and 20 ng/mL soluble recombinant IL-4 (BioLegend 574004). Cells were then spun down at 500xg for 5 min, resuspended in PBS and plated in 96-well round bottom plates. Cells were stained with LIVE/DEAD Fixable Blue Dead Cell Stain in PBS at room temperature for 15 min protected from light. Cells were spun down and resuspended in Fluorescence-activated cell sorting (FACS) buffer (PBS + 2% FBS + 0.02% Sodium Azide) supplemented with CellBlox Blocking Buffer, rat/hamster/mouse serum, Brilliant Staining Buffer, anti-CD20 BUV805, anti-CD86 Super Bright 436, anti-CD45 AlexaFluor 532 (Thermofisher #58-0459-42), anti-CD14 NovaFluor Blue 585 (Thermofisher #H019T03B04), anti-CD3 NovaFluor Blue 585 (Thermofisher #H002T03B04), anti-CD40 PE-Dazzle 594 (Biolegend #334342), anti-CD19 NovaFluor Red 700(Thermofisher #H004T03R03), and anti-CD69 FITC(Biolegend #310904). Cells were incubated in staining buffer for 30 min at room temperature protected from the light. Cells were then washed with FACS buffer and fixed with BD FACS Lysing Solution per manufacturer’s instructions. Fixed cells were resuspended in PBS and data were acquired on a Cytek Aurora. Data were analyzed using FlowJo and SpectroFlo software. Geometric Mean Fluorescence Intensity of samples were normalized to EtOH control group and statistical significance was determined using a one-sample T-test with a theoretical value 1.

### Membrane order versus disorder measurement

B cells were washed and resuspended in serum free RPMI without phenol red. Cells were treated with designated lipid or 0.009% EtOH loading control for 10 min at 37 °C. Cells were then stained with 1 uM Di-4ANEPPDHQ and mean fluorescence was analyzed using an Accuri C6 Flow Cytometer. “Ordered” membrane was read in FL1 channel and “disordered” membrane was read in the FL3 channel. Data is presented as a fold change normalized to EtOH set to 1. Data was compiled from 3 individual experiments with distinct donors. Statistical significance was determined using One Sample T-test with theoretical value 1.

### Immunoblotting

Primary human B cells were treated with various concentrations of GML or 0.009% EtOH as a vehicle control in serum free RPMI 1640 media. Cells were then stimulated for 10 min with anti-IgM (Jackson ImmunoReserach 109-006-129) and recombinant IL-4 (BioLegend 574004). Cells were lysed with 2 × sample buffer, heated to 95 °C, and sonicated. Cell lysates were separated across a 4–15% polyacrylamide gel via gel electrophoresis and transferred to PVDF. The membranes were blocked with SEA BLOCK blocking buffer (ThermoFisher) diluted in PBS and incubated overnight at 4 °C with primary antibody. After washing, samples were incubated with secondary antibodies for 30 min at room temperature and then imaged and quantified using a Licor Odyssey. Samples were normalized to GAPDH and analyzed as a fold change with stimulated EtOH control set to 1. Data presented is 3 experimental replicates and analyzed using One-Sample T-test with theoretical value 1.

### Total internal reflection fluorescence microscopy

Primary B cells were treated with 10 ug/mL of GML, or 0.009% EtOH control and stimulated for 10 min with plate-bound anti-IgM antibody in serum-free media without phenol red on a cover glass 4-well chamber slide. Cells were then fixed with 4% paraformaldehyde for 30 min, permeabilized using 0.25% Triton X-100 for 5 min, blocked with SEA BLOCK blocking buffer diluted with PBS, and then coated with appropriate reagents: CD19 (Invitrogen 4349181), Total AKT (Cell signaling), pBLNK Tyr96 (Bios bs-3054R), phosphor-CD79A (Cell Signaling) and actin was stained with tetramethylrhodamine (TMR)-phalloidin (Sigma-Aldrich). Cluster formation was quantified in ImageJ by measuring Mean Pixel Intensity along a straight line across the widest part of the cell. Filopodia formation was analyzed by counting number of filopodia per cell in addition to measuring the length of filopodia using the ImageJ measuring tool. Student’s T-test was used to calculate statistical significance.

## Supplementary Information

Supplementary Information.

## Data Availability

All data generated during this study are available from the corresponding author on reasonable request.

## Abbreviations

GML: Glycerol monolaurate
BCR: B cell receptor
BLNK: B cell linker protein
IL-4: Interleukin-4
IL-6: Interleukin-6
TNFα: Tumor necrosis factor-alpha

## References

[1] Schlievert PM (1992). Effect of glycerol monolaurate on bacterial growth and toxin production. Antimicrob. Agents Chemother..

[2] Projan SJ (1994). Glycerol monolaurate inhibits the production of beta-lactamase, toxic shock toxin-1, and other staphylococcal exoproteins by interfering with signal transduction. J. Bacteriol..

[3] Peterson ML, Schlievert PM (2006). Glycerol monolaurate inhibits the effects of gram-positive select agents on eukaryotic cells. Biochemistry.

[4] Strandberg KL (2010). Glycerol monolaurate inhibits Candida and Gardnerella vaginalis in vitro and in vivo but not Lactobacillus. Antimicrob. Agents Chemother..

[5] Schlievert PM, Peterson ML (2012). Glycerol monolaurate antibacterial activity in broth and biofilm cultures. PLoS ONE.

[6] Mueller EA, Schlievert PM (2015). Non-aqueous glycerol monolaurate gel exhibits antibacterial and anti-biofilm activity against gram-positive and gram-negative pathogens. PLoS ONE.

[7] Schlievert PM (2019). Glycerol monolaurate contributes to the antimicrobial and anti-inflammatory activity of human milk. Sci. Rep..

[8] Liau KM (2011). An open-label pilot study to assess the efficacy and safety of virgin coconut oil in reducing visceral adiposity. ISRN Pharmacol..

[9] Li Q (2009). Glycerol monolaurate prevents mucosal SIV transmission. Nature.

[10] Lin YC (2009). Glycerol monolaurate and dodecylglycerol effects on staphylococcus aureus and toxic shock syndrome toxin-1 in vitro and in vivo. PLoS ONE.

[11] Witcher KJ, Novick RP, Schlievert PM (1996). Modulation of immune cell proliferation by glycerol monolaurate. Clin. Diagn. Lab. Immunol..

[12] Zhang MS, Sandouk A, Houtman JC (2016). Glycerol monolaurate (GML) inhibits human T cell signaling and function by disrupting lipid dynamics. Sci. Rep..

[13] Zhang MS (2018). Glycerol monolaurate induces filopodia formation by disrupting the association between LAT and SLP-76 microclusters. Sci. Signal.

[14] Fosdick MG (2021). Suppression of human T cell activation by derivatives of glycerol monolaurate. Sci. Rep..

[15] Tanaka S, Baba Y, Wang J-Y (2020). B Cell receptor signaling. B Cells in immunity and tolerance.

[16] Clark EA, Shu G (1990). Association between IL-6 and CD40 signaling. IL-6 induces phosphorylation of CD40 receptors. J. Immunol..

[17] Burington B (2011). CD40 pathway activation status predicts response to CD40 therapy in diffuse large B cell lymphoma. Sci. Transl. Med..

[18] Jeannin P (1997). CD86 (B7–2) on human B cells: a functional role in proliferation and selective differentiation into IgE- AND IgG4-producing cells*. J. Biol. Chem..

[19] Gupta N, DeFranco AL (2007). Lipid rafts and B cell signaling. Semin. Cell Dev. Biol..

[20] Axelsson S (2020). A combination of the activation marker CD86 and the immune checkpoint marker B and T lymphocyte attenuator (BTLA) indicates a putative permissive activation state of B cell subtypes in healthy blood donors independent of age and sex. BMC Immunol..

[21] de Boer M (1993). Ligation of B7 with CD28/CTLA-4 on T cells results in CD40 ligand expression, interleukin-4 secretion and efficient help for antibody production by B cells. Eur. J. Immunol..

[22] Freeman GJ (1995). B7–1 and B7–2 do not deliver identical costimulatory signals, since B7–2 but not B7–1 preferentially costimulates the initial production of IL-4. Immunity.

[23] Ollig J (2019). B cell activation and proliferation increase intracellular zinc levels. J. Nutr. Biochem..

[24] Vazquez BN (2009). CD69 gene is differentially regulated in T and B cells by evolutionarily conserved promoter-distal elements. J. Immunol. (Baltimore, Md: 1950).

[25] Craston R (1997). Temporal dynamics of CD69 expression on lymphoid cells. J. Immunol. Methods.

[26] Bolger-Munro M (2019). Arp2/3 complex-driven spatial patterning of the BCR enhances immune synapse formation, BCR signaling and B cell activation. Elife.

[27] Li JW (2019). The coordination between B cell receptor signaling and the actin cytoskeleton during B cell activation. Front. Immunol..

[28] Treanor B (2010). The membrane skeleton controls diffusion dynamics and signaling through the B cell receptor. Immunity.

[29] Cheng PC (1999). A role for lipid rafts in B cell antigen receptor signaling and antigen targeting. J. Exp. Med..

[30] Stone MB (2017). Protein sorting by lipid phase-like domains supports emergent signaling function in B lymphocyte plasma membranes. Elife.

[31] Maity PC (2015). The nanoscale organization of the B lymphocyte membrane. Biochem. Biophys. Acta..

[32] Wong J (2000). Functional complementation of BLNK by SLP-76 and LAT linker proteins. J. Biol. Chem..

[33] Fujimoto M (2000). CD19 regulates Src Family protein tyrosine kinase activation in B lymphocytes through processive amplification. Immunity.

[34] Otero DC, Omori SA, Rickert RC (2001). CD19-dependent activation of Akt Kinase in B-lymphocytes*. J. Biol. Chem..

[35] Wilson BS, Pfeiffer JR, Oliver JM (2002). FcepsilonRI signaling observed from the inside of the mast cell membrane. Mol. Immunol..

[36] Pierce SK (2002). Lipid rafts and B-cell activation. Nat. Rev. Immunol..

[37] Kimachi K, Sugie K, Grey HM (2003). Effector T cells have a lower ligand affinity threshold for activation than naive T cells. Int. Immunol..

[38] Kumar R (2011). Increased sensitivity of antigen-experienced T cells through the enrichment of oligomeric T cell receptor complexes. Immunity.

[39] Sproul TW (2000). Cutting edge: B Cell antigen receptor signaling occurs outside lipid rafts in immature B cells. J. Immunol..

[40] Chung JB, Baumeister MA, Monroe JG (2001). Cutting Edge: differential sequestration of plasma membrane-associated B cell antigen receptor in mature and immature B cells into glycosphingolipid-enriched domains. J. Immunol..

[41] Tani-ichi S (2005). Structure and function of lipid rafts in human activated T cells. Int. Immunol..

[42] Cho J-H (2010). T cell receptor-dependent regulation of lipid rafts controls naive CD8+ T cell homeostasis. Immunity.

