# Supplementary figures and images for: Glycerol monolaurate inhibition of human B cell activation
Source: Sci Rep. 2022 Aug 5;12:13506. doi: 10.1038/s41598-022-17432-4 (PMC9355977; doi:10.1038/s41598-022-17432-4)

Supplemental Figure 1

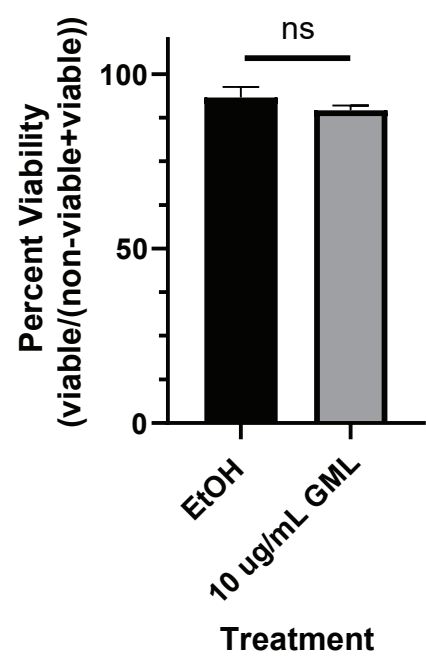

Supplemental Figure 2

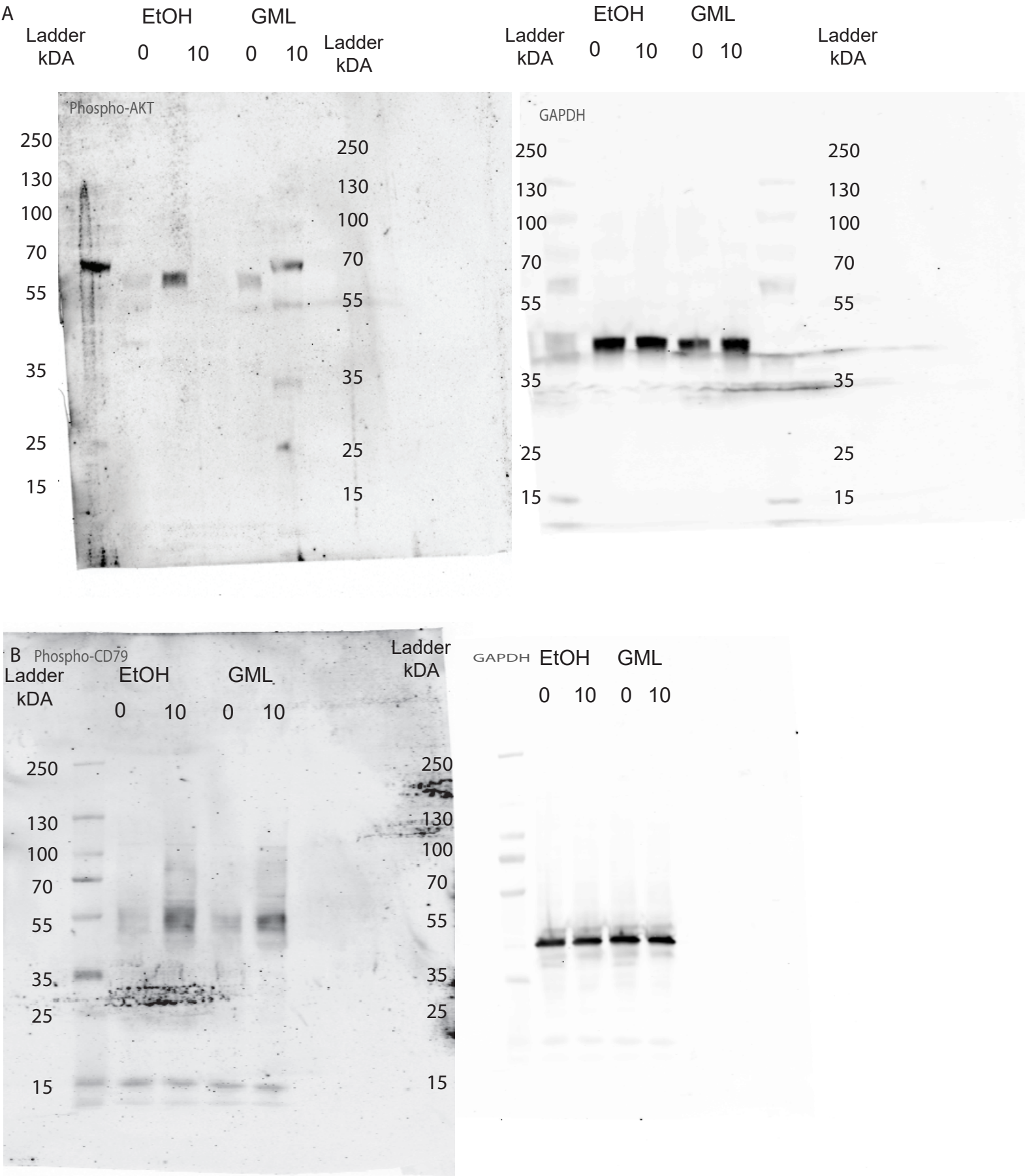

Supplement: Supplementary file 1 — Supplementary Information. [file 41598_2022_17432_MOESM1_ESM.pdf]
